# Replacement of fluoroscopy by ultrasonography in the evaluation of hemidiaphragm function, an exploratory prospective study

**DOI:** 10.1186/s13089-023-00355-0

**Published:** 2024-01-08

**Authors:** Søren Helbo Skaarup, Peter Juhl-Olsen, Anne Sofie Grundahl, Brian Bridal Løgstrup

**Affiliations:** 1 Department of Respiratory Medicine and Allergy, Aarhus University Hospital, Palle Juul-Jensens Boulevard 99, 8200 Aarhus, Denmark; 2 Department of Cardiothoracic and Vascular Surgery, Anaesthesia Section, Aarhus University Hospital, Aarhus, Denmark; 3 Department of Emergency Medicine, Randers Regional Hospital, Randers, Denmark; 4 Department of Cardiology, Aarhus University Hospital, Aarhus, Denmark

## Abstract

**Introduction:**

Dysfunction of the diaphragm may ultimately lead to respiratory insufficiency and compromise patient outcome. Evaluation of diaphragm function is cumbersome. Fluoroscopy has been the gold standard to measure diaphragmatic excursion. Ultrasonography can visualize diaphragm excursion and holds many advantages such as no radiation exposure, increased portability and accessibility. However, correlation between fluoroscopy and ultrasonography has never been studied. We aimed to compare fluoroscopic and ultrasound measures of diaphragm excursion to determine if ultrasonography can replace fluoroscopy.

**Methods:**

We performed ultrasound and fluoroscopy simultaneously during sniff inspiration and at total inspiratory capacity in patients with chronic obstructive pulmonary disease, heart failure and in healthy volunteers. Cranio-caudal excursion was measured by fluoroscopy and compared directly to M-mode excursion, B-mode excursion, area change, resting thickness, thickening fraction and contraction velocity measured by ultrasonography.

**Results:**

Forty-two participants were included. The Pearson correlation between M-mode and fluoroscopy excursion was 0.61. The slope was 0.9 (90%CI 0.76–1.04) in a regression analysis. Using the Bland–Altman method, the bias was − 0.39 cm (95% CI − 1.04–0.26), *p* = 0.24. The Pearson correlation between fluoroscopy and B-mode and area change ultrasonography was high; low for thickness and fraction. All correlations were lower during sniff inspiration compared with inspiratory capacity breathing.

**Conclusion:**

Ultrasonography has an acceptable correlation and bias compared to fluoroscopy and can thus be used as the primary tool to evaluate diaphragm excursion.

**Supplementary Information:**

The online version contains supplementary material available at 10.1186/s13089-023-00355-0.

## Introduction

The diaphragm plays a pivotal role in maintaining adequate ventilation. Dysfunction of the diaphragm may ultimately lead to respiratory insufficiency and compromise patient outcome. A multitude of diseases may contribute to diaphragm dysfunction, spanning from neurological lesions and phrenic nerve disorders to neuromuscular junction dysfunction, thoracic surgical interventions, and pulmonary and pleural diseases. [1–3] Thus, assessment of diaphragm function is highly relevant for a diverse array of patients across various medical specialties, including neurology, emergency and intensive care medicine, pulmonology, cardiology, thoracic surgery, and anaesthesiology [1].

Evaluation of diaphragm function remains a challenge and consensus is lacking on an optimal evaluation method [1, 4–6]. Various approaches, including neuromuscular assessment as well as static and dynamic imaging methods such as fluoroscopy and ultrasonography, have been employed. Phrenic nerve stimulation is cumbersome, risky, and uncomfortable for patients, limiting widespread use in clinical practice. Chest X-ray and computed tomography of the chest can identify unilateral or bilateral diaphragm elevation, but fail to provide insights into diaphragm motion or contractility. Fluoroscopy has been extensively used and is often regarded as the gold standard for assessment of diaphragm function. Recently, ultrasonography has gained prominence as a viable alternative. Multiple ultrasound methods have been developed to assess not only diaphragmatic excursion using M-mode, cranio-caudal excursion, and area change, but also contractile function by measuring diaphragmatic thickness, thickening fraction, and contraction velocity [7–14]. Ultrasonography offers improved accessibility and thus a preferred clinical choice. Ultrasonography use has expanded beyond the intensive care unit, where it assists in weaning patients from mechanical ventilation [15–17], to the pulmonary procedural room for thoracentesis [18, 19], and even in emergency settings to identify causes of respiratory failure [20, 21].

While previous studies have demonstrated good inter- and intra-observer agreement and acceptable correlations between ultrasound methods and spirometry values, the comparison between fluoroscopy and ultrasonography has not been thoroughly explored. This comparison needs to be addressed to qualify whether ultrasound potentially can replace fluoroscopy as the primary evaluation method of the diaphragm, which was the hypothesis of this study. Patients in who fluoroscopy are difficultly performed, like in intensive care, during pleural procedures or in emergency situation may especially benefit from such a replacement. Accordingly, we simultaneously performed fluoroscopy and ultrasonography to assess diaphragm motion and contractility in patients and volunteers, respectively, to evaluate correlations between the two methods.

## Methods

### Study design

This observational study compared fluoroscopy to multiple ultrasound methods for evaluation of diaphragm function reporting level of agreement.

The study was approved by local ethics committee (journal no. 1-10-72-104-19) and registered at Clinicaltrials.org (NCT04098939). Data were stored in REDCap hosted by Aarhus University.

### Participants

Participants were recruited from Department of Respiratory Diseases and Allergy, Department of Cardiology and from the Clinic for long-Covid symptoms at Aarhus University Hospital, Denmark.

Eligible individuals provided written, informed consent prior to inclusion, and had either chronic obstructive pulmonary disease (COPD), interstitial lung disease (ILD), had undergone thoracic surgery (heart transplantation or a left ventricular assist device (LVAD) implant), had suffered COVID-19 infection or were healthy volunteers. Individuals with known diaphragm dysfunction due to neuromuscular disease, pleural effusion or pneumothorax were excluded.

### Setting

All examinations were performed during a single visit. Baseline characteristics were registered and spirometry was performed including measurement of functional vital capacity, forced expiratory volume at one second and the ratio between these. Then, the simultaneously fluoroscopic and ultrasound measures were recorded with patients in upright sitting position and stored. All examinations were performed in a cardiac laboratory room with access to c-arm fluoroscopy and ultrasonography (Logiq S8, GE Healthcare, USA).

### Recordings

A total of ten diaphragm recordings were made in each participant and followed international accepted standards [1]. Film clips were stored for future analyses.

**Fig. 1:**
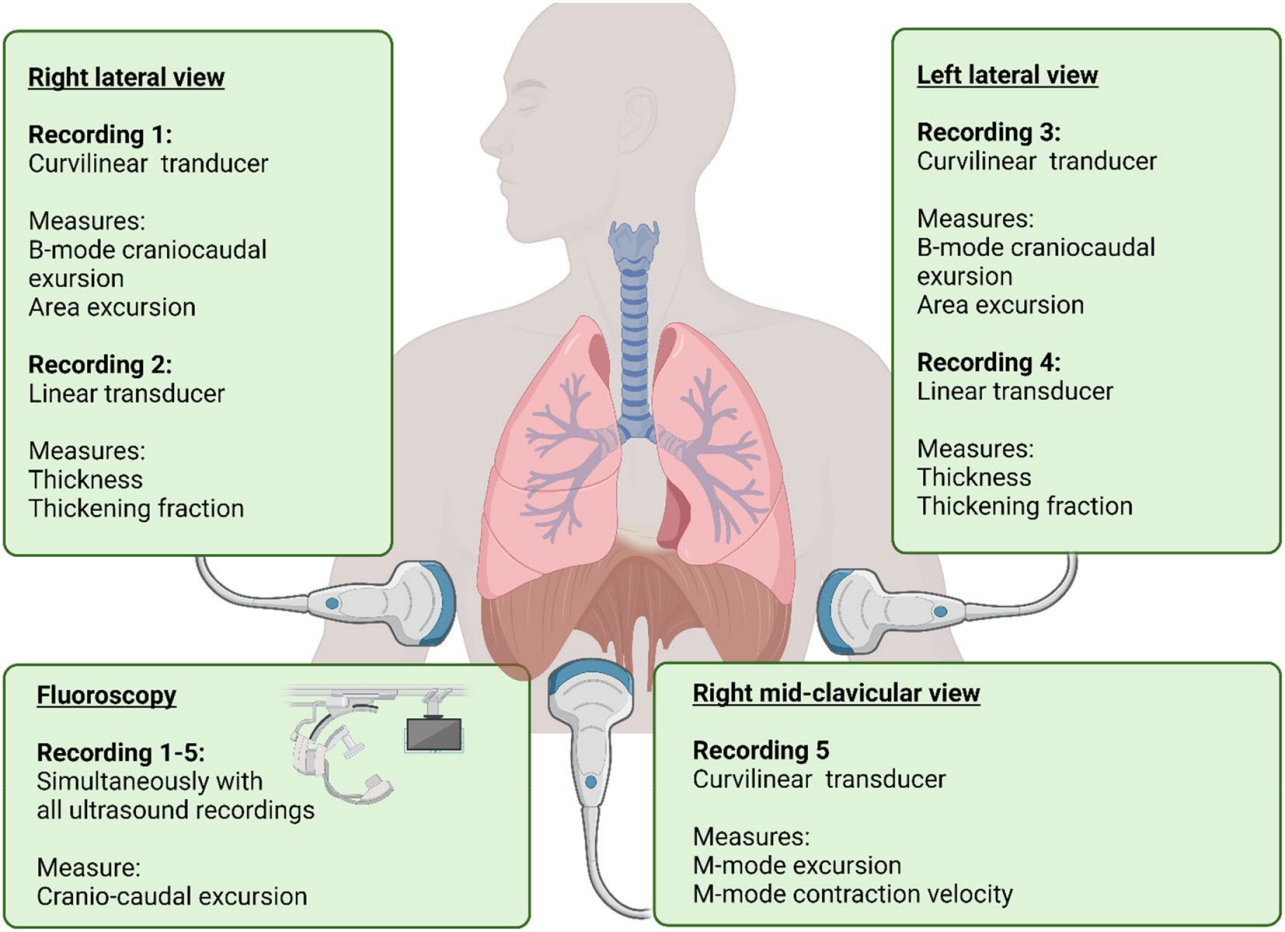
Study setup. With participants in sitting position, ultrasound and fluoroscopic recordings were made simultaneously. Ultrasound recorded diaphragm excursion from the left lateral view (recording 1 and 2 with curvilinear and linear transducer), from the right lateral view (recording 3 and 4 with curvilinear and linear transducer), and from the midclavicular view (recording 5 with curvilinear transducer). All recordings were made in both sniff and IC inspiration, a total of ten recordings. Fluoroscopy recorded diaphragm excursion in the same breathing manoeuvre as was recorded by ultrasonography. The recordings were later analyzed and the diaphragm excursion was measured as described in the figure and in the text

Four ultrasound and fluoroscopy recordings were performed simultaneously in the left lateral position, two with a curvilinear ultrasound transducer (3–5 Hz, GE Healthcare, USA) using the spleen as the acoustic window, and two with a linear transducer (7–11 Hz, GE Healthcare, USA) in the zone of apposition. Similarly, four ultrasound and fluoroscopy recordings were performed simultaneously in the right lateral position, two with the curvilinear ultrasound transducer using the liver as the acoustic window, and two with the linear transducer in the right zone of apposition. Finally, two ultrasound and fluoroscopy recordings were performed simultaneously with the curvilinear transducer in the right mid-clavicular position. Recordings are illustrated in Fig. 1.

### Breathing manoeuvres

All recordings were made during two simple breathing manoeuvres in sequence. First, ultrasound and fluoroscopy recordings were performed simultaneously while the participants performed a slow inspiration from resting respiration to maximal inspiration (inspiratory capacity (IC)). Second, recordings were repeated while the participants did a voluntary quick forced sniff inspiration. Fluoroscopy and ultrasound recordings were performed during the same breathing manoeuvre.

### Fluoroscopy measures

Fluoroscopy recordings were analyzed in XERO Viewer 8.1.1 (using full fidelity showing). The cranio-caudal excursion of the diaphragm was measured as the distance of the hemidiaphragm top point from expiration to the lowest point during inspiration in centimetres for both the IC and the sniff inspiration.

### Ultrasound measures

Ultrasound recordings were analyzed using ultrasonography equipment.

Three ultrasound measures were quantified in the lateral position of the left and the right hemidiaphragm, respectively:

- Excursion: The cranio-caudal excursion of the top of the hemidiaphragm was measured in B-mode (2D-mode) in centimetres according to Houston et al. [7]
- Area Change: The change in intrathoracic area between end-expiration and end-inspiration was measured in square centimetres (cm.^2^) by tracking the curve of the hemidiaphragm according to Skaarup et al. [9]
- Thickness and thickening fraction: Hemidiaphragm thickness was measured in centimetres in maximal inspiration and expiration in the zone of apposition, where the diaphragm was identified as a layered structure on top of the liver and spleen. Thickening fraction was calculated [1, 22].

Two ultrasound measures were quantified in the midclavicular position:

- M-mode excursion: A motion-mode (M-mode) line was positioned at the posterior part of the right hemidiaphragm and excursion along the M-mode line, toward the transducer was measured in centimetres [10, 11, 23].
- Contraction velocity: The time from expiration to inspiration was measured in seconds on the M-mode curve and contraction velocity was calculated by the ultrasonography in centimetres per second [24].

### Statistical methods

Demographic data and results from spirometry, ultrasound and fluoroscopy are presented in means, standard deviations (SD) and percentages as appropriate. Normal data distribution was assessed by histograms, quantile–quantile plots, and Shapiro–Wilk tests. Repeated measurements were analyzed by a multivariate repeated measurements model. Missing observations were presumed missing at complete random, and no imputation was made. All data were analyzed using STATA version 14 (Texas, USA). Two-sided *p*-values < 0.05 were considered statistically significant.

### Comparisons

Ultrasound M-mode excursion of the right hemidiaphragm recorded in the midclavicular position was compared to fluoroscopy excursion using the Bland–Altman method, a linear regression analysis and Pearson’s correlation were used in both IC and sniff inspiration. [25, 26] Fluoroscopy was considered as the gold standard and the M-mode excursion was compared to this.

In the lateral ultrasound positions measures of right and left hemidiaphragm excursion were compared to fluoroscopy excursion with linear regression and Pearson’s correlation. The Bland–Altman method was applied to 2D measures only, as the unit for the area change (cm^2^) was different from excursion measures and thus did not allow for Bland–Altman analysis.

Accordingly, diaphragm thickness, thickening fraction and contraction velocity were compared to fluoroscopy using linear regression and Pearson´s correlation.

Finally, ultrasound and fluoroscopic measures were compared to spirometry measures of FVC and FEV1 using linear regression and Pearson´s correlation.

## Results

### Participants

A total of 42 participants were included in the study. Eighteen participants were recruited from Department of Respiratory Diseases and Allergy, 13 participants from Department of Cardiology and 11 participants had had COVID-19 or were healthy volunteers. Participant characteristics are shown in Table 1.

**Table 1:** Demographic data on study participants (*n* = 42)

	Total		Respiratory disease		Heart disease		Covid or healthy	
Variable	Mean	Standard deviation	Mean	Standard deviation	Mean	Standard deviation	Mean	Standard deviation
Sex, % women	15 (37%)		7 (39%)		2 (15%)		6 (55%)	
Age (years)	59.7	13.3	64.3	6.5	57.5	16.8	53	0
Height, cm	176.7	10.8	172.9	10.3	182.3	9,6	176	11,3
Weight, kg	81.9	17.4	71.9	12.8	92.3	16	87.5	17.5
FEV1, l/min	1.7	1.1	0.9	0.4	2.6	0.9	2.6	0.8
FEV1, % of expected	50	28	32.3	15.8	75.3	14.9	95.5	4.9
FVC, l	3.1	1.2	2.5	0.9	3.7	1.1	3.5	1
FVC, % of expected	79	23	71.4	22.8	88.1	15.6	105	18.3
Ratio, FVC/FEV1	0.5	0.2	0.4	0.1	0.7	0.1	0.72	0.04

*FEV1* Forced expiratory volume after one second. *FVC* Forced vital capacity

### Overall fluoroscopy results

The mean diaphragm excursion was 4.3 cm (SD ± 2.1) on fluoroscopy in the five repeated IC inspiration manoeuvres. The excursion was 4.0 cm (SD ± 2.2) at the right hemidiaphragm and 4.8 cm (SD ± 2.1) at the left hemidiaphragm. For the five repeated sniff inspirations, the overall mean diaphragm excursion was 1.9 cm (SD ± 1.0), 1.7 cm (SD ± 1.2) at the right hemidiaphragm and 2.1 cm (SD ± 1.1) at the left hemidiaphragm.

### Overall ultrasonography results

At the right hemidiaphragm, M-mode excursion in the midclavicular line was 4.3 cm (SD ± 1.7) in IC inspiration. For the right lateral ultrasound position the area change was 36.4 cm^2^ (SD ± 22) and the B-mode excursion was 3.1 cm (SD ± 1.8). Resting thickness was 0.4 cm (SD ± 0.1) and the thickening fraction was 0.3 (SD ± 0.2). During the sniff inspiration, the right hemidiaphragm M-mode excursion was 3.3 cm (SD ± 1.1) and the contraction velocity was 7.9 cm/sec (SD ± 4.5). The area change was 26.1 cm2 (SD ± 13.5).

For the left hemidiaphragm position, the area change was 36.7 cm2 (SD ± 22) during IC inspiration. B-mode excursion was 3.1 cm (SD ± 2.1). Left hemidiaphragm resting thickness was 0.4 cm (SD ± 0.1) and thickening fraction was 0.24 (SD ± 0.2). During the sniff inspiration the left area change was 33.5 cm2 (SD ± 24).

### Comparison of excursion with fluoroscopy and M-mode ultrasonography in the midclavicular position

#### IC inspiration manoeuvre

**Fig. 2:**
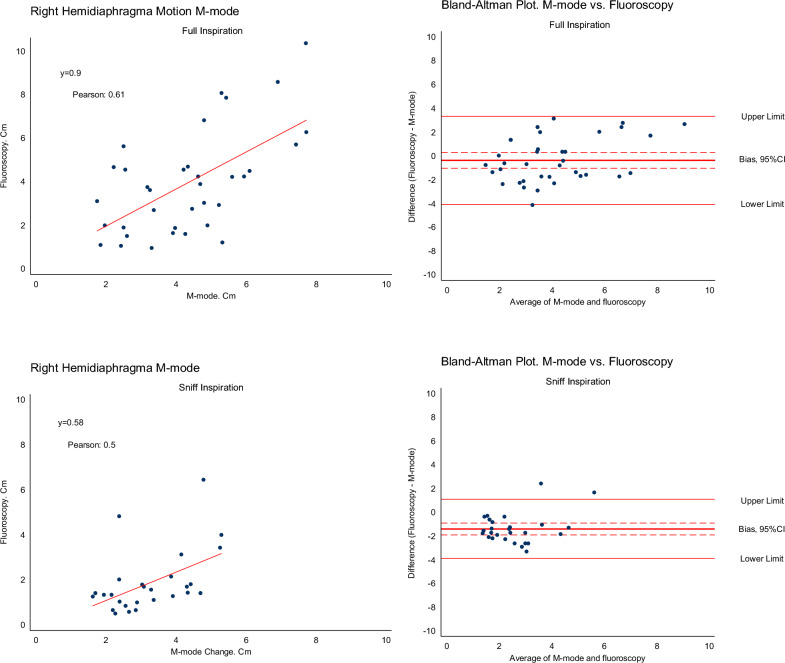
Linear regression between right hemidiaphragm excursion measured with fluoroscopy and ultrasonography by M-mode for inspiratory capacity inspiration and sniff inspiration manoeuvres (left side). Bland–Altman plots of the bias between M-mode and fluoroscopy versus average of M-mode and fluoroscopy for inspiratory capacity inspiration and sniff inspiration manoeuvres (right side) with 95% limits of agreement. See main text for details

Linear regression analysis revealed a slope at 0.9 (95% CI 0.8–1.0), *p* < 0.001 (Fig. 2).

The mean fluoroscopic excursion on the recordings performed simultaneously with the ultrasound M-mode excursion was 4.1 cm (SD ± 2.3) and the corresponding mean M-mode excursion was 4.3 cm (SD ± 1.7) with a mean difference on − 0.2 cm (95% CI − 1.1–0.7); *p* = 0.69, including all observations (*n* = 40). The right hemidiaphragm motion was lower when measured by fluoroscopy than by M-Mode in 60% of the observations. A Bland–Altman plot showed a mean bias of -0.4 cm (95% CI − 1.0–0.3); p = 0.24, when only including observation where both measurements were available for comparison (*n* = 35) (Fig. 2). The upper limit of agreement was 3.3 cm and lower limit was − 4.1 cm. A histogram of fluoroscopy and M-mode ultrasound, Additional file 1: Fig. S1, showed parametric distribution. The Pearson correlation coefficient between M-mode ultrasonography and fluoroscopy was 0.61.

#### Sniff inspiration manoeuvre

A linear regression analysis showed a slope at 0.57 (95% CI 0.44–0.72), *p* < 0.001 (Fig. 2). The mean right hemidiaphragm motion was 1.8 cm (SD ± 1.2) when measured with fluoroscopy and 3.3 cm (SD ± 1.1) with M-mode ultrasonography, resulting in a mean difference of -1.5 cm (95% CI − 2.1–− 0.9), *p* < 0.001, *n* = 39. In the Bland–Altman plot shown in Fig. 2, the bias between fluoroscopy and ultrasonography was -1.4 cm (95% CI − 1.9 to − 0.9), *p* < 0.001, in observations where both measurements were available (*n* = 27). The upper limit of agreement was 1.1 cm and the lower limit was -3.9 cm. Spearman’s correlation was 0.52.

### Comparison of excursion with fluoroscopy and B-mode ultrasonography in the right and left hemidiaphragm positions

**Fig. 3:**
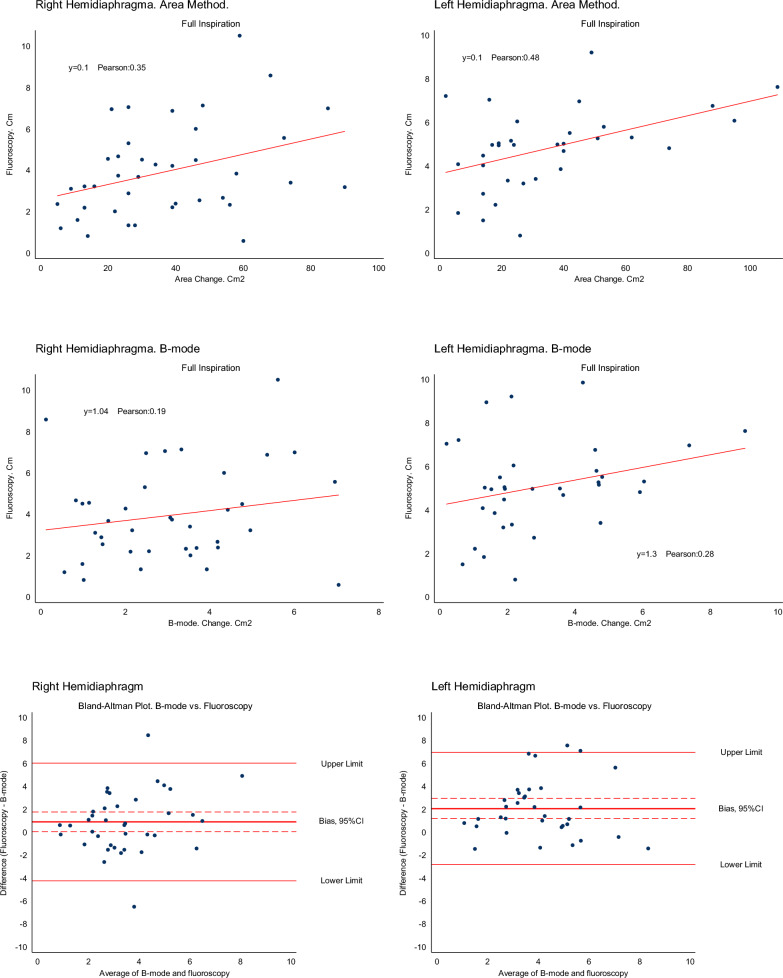
Correlations between fluoroscopy and right and left hemidiaphragm motion measured with ultrasonography by the Area method and the B-mode method and Bland–Altman plots display biases and 95% limits of agreement between B-mode and fluoroscopy

A linear regression analysis between right hemidiaphragm excursion and fluoroscopy showed a slope at 1.03 (95% CI 0.78–1.29), *p* < 0.001 (Fig. 3). The mean right hemidiaphragm excursion was 0.9 cm (95% CI 0.01–1.8), *p* = 0.05, *n* = 41, higher when measured by fluoroscopy than by B-mode ultrasonography. The mean bias for the right hemidiaphragm excursion was 0.9 cm (95% CI 1.2–3.0), *p* < 0.001, n = 0.38, and upper and lower limits of agreement were 6.0 cm and − 4.2 cm as shown in the Bland–Altman plot in Fig. 3. Spearman’s correlation was 0.15.

Similar comparisons on the left hemidiaphragm found a slope at 1.2 (c 1.0–1.6), a mean difference of 1.8 cm (95% 0.8–2.7), *p* < 0.001, *n* = 41, and a Spearman’s correlation of 0.26. The bias between left fluoroscopy and B-mode ultrasonography was 2.1 cm (95% CI 1.2–2.9), *p* < 0.001, *n* = 32 with upper and lower limits of agreement of 7.0 cm and -2.8 cm as shown in the Bland–Altman plot in Fig. 3.

### Comparison of fluoroscopy and the Area method in the right and left hemidiaphragm positions

The linear regression coefficient of left hemidiaphragm area change during IC inspiration was 0.1 (94%CI 0.08–0.12), *p* < 0.001, and the Pearson correlation coefficient was 0.48. For the right hemidiaphragm, the linear regression coefficient was 0.1 (95% CI 0.07–0.11), *p* < 0.001, and the Pearson correlation was 0.34 (Fig. 3).

During the sniff inspiration manoeuvre the linear regression coefficient was 0.04 (95% CI 0.03–0.05), *p* < 0.001 with a Pearson correlation coefficient of 0.20 for the left hemidiaphragm. The corresponding linear regression coefficient was 0.05 (95% CI 0.03–0.07), *p* < 0.001, and the Pearson correlation coefficient was 0.02 for the right hemidiaphragm.

### Comparison of diaphragm thickness fraction and fluoroscopy excursion

There were no significant linear regression coefficients for the right hemidiaphragm, -0.8 (95% CI -4.14–2.47), *p* = 0.61, and the Pearson correlation coefficient was -0.08, or on the left hemidiaphragm, − 2.01 (95% CI − 5.7–1.7), *p* = 0.27, the Pearson correlation coefficient was -0.18 between thickness fractions and fluoroscopy measurements of diaphragm excursion.

### Comparison of fluoroscopic and ultrasound measures of diaphragm excursion to dynamic lung function measures

Correlations between dynamic lung function values of FVC and FEV1, and fluoroscopy and M-mode ultrasonography of diaphragm excursion are listed in Table 2.

**Table 2:** Linear correlation coefficients and Pearson correlation coefficients of M-mode ultrasound-and fluoroscopic measures of the right hemidiaphragm excursion during maximal inspiration capacity inspiration and sniff inspiration

Right hemidiaphragm	Midclavicular view	Linear regression						Pearson correlation coefficient
		Coef	Std	*t*	*p*-value	95% CI		
FEV1								
IC inspiration	Fluoroscopy	0.21	0.07	2.89	0.01	0.06	0.35	0.57
	M-mode	0.26	0.11	2.46	0.02	0.04	0.48	0.53
Sniff inspiration	Fluoroscopy	0.24	0.15	1.58	0.13	− 0.07	0.54	0.42
	M-mode	0.38	0.23	1.63	0.12	− 0.11	0.87	0.50
	Velocity	0.10	0.08	1.33	0.20	− 0.06	0.27	0.41
FVC								
IC inspiration	Fluoroscopy	0.23	0.08	2.94	0.01	0.07	0.39	0.60
	M-mode	0.19	0.12	1.51	0.14	− 0.07	0.44	0.55
Sniff inspiration	Fluoroscopy	0.18	0.17	1.05	0.30	− 0.17	0.52	0.49
	M-mode	0.41	0.23	1.77	0.10	− 0.08	0.91	0.27
	Velocity	0.12	0.09	1.35	0.20	− 0.06	0.30	0.33

*CI* Confidence interval, *FEV1* Forced expiratory volume in one second, *FVC* Forced vital capacity, *Std.* Standard deviation

Linear regression coefficients and Pearson correlation coefficients between fluoroscopic and ultrasound measures of each hemidiaphragm excursion are listed in Table 3. Coefficients and correlations were higher for measures at the left hemidiaphragm than at the right. Correlation and coefficients between FEV1 and FVC and resting diaphragm thickness and thickening fraction were poor and statistically insignificant (Table 3).

**Table 3:** Linear coefficient and Pearson correlation coefficient of each hemidiaphragm motion measured by fluoroscopy, area change, resting (end expiratory) thickness and thickening fraction compared to spirometry results

Both hemidiaphragms	Lateral views	Linear regression						Pearson correlation coefficient
		Coef.	Std.	*t*	*p*-value	95% CI		
FEV1								
Left hemidiaphragm	Fluoroscopy	0.28	0.07	3.76	0.001	0.13	0.43	0.7369
	Ultrasonography	0.04	0.01	2.81	0.01	0.01	0.07	0.5285
Right hemidiaphragm	Fluoroscopy	0.20	0.07	2.64	0.01	0.04	0.35	0.4575
	Ultrasonography	0.02	0.01	2.73	0.01	0.01	0.04	0.4598
FVC								
Left hemidiaphragm	Fluoroscopy	0.31	0.08	3.86	0.001	0.15	0.48	0.7332
	Ultrasonography	0.05	0.01	4.13	0.001	0.03	0.08	0.6913
Right hemidiaphragm	Fluoroscopy	0.19	0.08	2.27	0.03	0.02	0.36	0.3992
	Ultrasonography	0.02	0.01	2.75	0.01	0.01	0.04	0.4731
FEV1								
Left hemidiaphragm	Resting thickness	− 6.75	3.78	− 1.79	0.09	− 14.5	1.03	− 0.34
	Thickening fraction	− 0.96	1.08	− 0.89	0.38	− 3.17	1.26	− 0.17
Right hemidiaphragm	Resting thickness	− 0.63	2.77	− 0.23	0.82	− 6.31	5.05	0.01
	Thickening fraction	0.04	0.77	0.05	0.96	− 1.54	1.62	− 0.0428
FEV								
Left hemidiaphragm	Resting thickness	− 6.01	4.31	− 1.40	0.18	− 14.9	2.86	− 0.2796
	Thickening fraction	− 0.30	1.22	− 0.24	0.81	− 2.80	2.21	− 0.0552
Right hemidiaphragm	Resting thickness	− 1.41	3.02	− 0.47	0.64	− 7.60	4.78	− 0.0715
	Thickening fraction	− 0.32	0.84	− 0.38	0.71	− 2.04	1.41	− 0.0507

*CI* Confidence interval, *FEV1* Forced expiratory volume in 1 s, *FVC* Forced vital capacity, *Std.* Standard deviation

### Repeatability of fluoroscopic measures

**Fig. 4:**
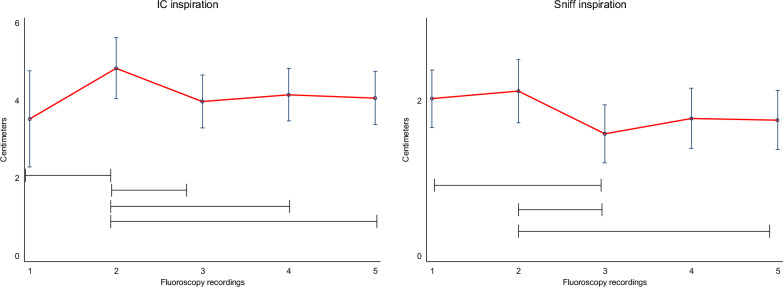
Repeated fluoroscopic measurements of right hemidiaphragm excursion in five consecutive measures of inspiration to full inspiration (to inspiratory capacity, IC) and as a sniff inspiration. During inspiratory capacity inspiration only one recording (the second) was statistically significantly different (*p* < 0.05) than the other measures. During sniff inspiration two (the second and the third) recordings were statistically different to the others (*p* < 0.05). Results indicate that reproducibility is lower in sniff inspiration than in inspiration to inspiratory capacity

Variation within the five-times repeated fluoroscopy measurements were significant during both the sniff inspiration and during IC as shown in Fig. 4 and in Additional file 1: Tables S1–S4.

## Discussion

This study aimed to compare diaphragm excursion with simultaneously performed fluoroscopic and ultrasound measurements. The results showed a high correlation between M-mode ultrasonography and fluoroscopy of the right hemidiaphragm during IC inspiration.

The diaphragm motion is the result of contraction of the muscular structures located in the periphery of the diaphragm dome. During contraction the diaphragm moves multi-directionally with both cranio-caudal and central-peripheral movements resulting in a complex motion that complicates accurate measurement of the excursion. Fluoroscopy measures the cranio-caudal excursion exclusively, while the M-mode ultrasonography in the midclavicular position measures the oblique excursion of the posterior part of the diaphragm towards the anterior of the abdomen. From the lateral positions, B-mode measures the cranio-caudal excursion while the Area method additionally measures change of the entire hemidiaphragm dome. Thus, although all methods measure the motion of the diaphragm, they capture different motion directions and the results are therefore probably not exactly the same.

Our results found a systematic difference between fluoroscopy and ultrasonography measurements; the difference was lower in M-mode than in B-mode. Based on the Bland–Altman analysis, the M-mode/fluoroscopy bias was well below any clinically relevant difference. The interpretation of this finding is that M-mode ultrasonography is interchangeable to fluoroscopy, at least when measures are made in IC inspiration. However, we found a high level of variation, which is likely due the different measurement angles of the excursion as mentioned above. The M-mode method is easily applied in the right mid-clavicular line where the liver serves as an acoustic window to the posterior part of the right hemidiaphragm. The M-mode technique has a much lower feasibility at the left hemidiaphragm, because the left midclavicular position is blocked by air in the abdomen, which limits acoustic access to the diaphragm [9]. Therefore, other methods to assess left hemidiaphragm motion using the spleen as reference with a lateral scanning point have been developed [7, 9, 11, 22]. In the lateral positions, M-mode is not applicable as the motion of the diaphragm is not perpendicular to the ultrasound transducer. The present study tested correlations between fluoroscopy and the lateral ultrasound access points by the Area method and the B-mode method. A higher Pearson correlation was found for the Area method than for the B-mode, especially at the left hemidiaphragm.

The biases between fluoroscopy and all ultrasonography measures were higher during the sniff inspiration than in IC inspiration. During the sniff inspiration, the rapid contraction of the circumferentially located musculature may not lead to a cranio-caudal excursion but only a quick excursion of the peripherally located muscular parts of the diaphragm. The M-mode method measures the motion of the posterior diaphragm where the muscle contraction occurs and fluoroscopy measures the motion of the central top of the diaphragm dome. This likely explains the difference. Likewise, when comparing ultrasound and the fluoroscopic measures to the dynamic lung function values, the correlation was higher for both ultrasonography and fluoroscopy in IC inspiration and low during sniff inspiration.

Similar, we compared fluoroscopy excursion to thickening fraction and contraction velocity and found low correlations. This is not surprising, because even though all of these indices measure diaphragm function, thickness, thickening fraction and velocity are markers of muscle contraction and not motion.

The study, however, has some limitations. First, although fluoroscopy and ultrasonography were performed simultaneously on the same breath to allow direct comparison of these methods, the dynamic lung function measurements were obtained at a different time. The setup was fairly challenging for the participants and performing spirometry at the same time as fluoroscopic and ultrasound recordings was not feasible. It was, therefore, not possible to measure the volume of air inhaled or exhaled during the breathing manoeuvres, which would have been interesting to compare fluoroscopy and ultrasonography variables. Instead, values from a subsequent spirometry were used as a comparator. These data were easily available, but the values may differ from the exact breathing manoeuvre where fluoroscopy and ultrasonography were performed. The study included patients with different causes for dyspnoea; COPD, heart failure and long-covid, and while diaphragm may have different function in these patients, the study was not powered to study difference in correlations between these disease categories. Furthermore, all participants were in the sitting position during the recordings. Diaphragm motion in the supine position may differ from the sitting position and results from this study may thus be less valid in intensive care patients or anaesthetised patients. Finally, we did not set a statistical definition on interchangeability between the methods. Interchangeability can be predefined if measurements are made on the exact same object. As mentioned above, the measurement angle differs between fluoroscopy and ultrasonography thus invalidating use of interchangeability statistical methods.

This study is the first to directly compare ultrasonography and fluoroscopy to quantify diaphragm excursion. Our results are an important contribution to define the optimal methodology for evaluation of diaphragm function [6, 27]. Ultrasound methods hold a number of advantages compared to fluoroscopy because of its versatility and accessibility. In many settings where evaluation of diaphragm motion is required, ultrasonography is already available as it is widely used in pulmonology, cardiology, anaesthesiology, intensive care and emergency medicine. Moreover, skills to perform ultrasound evaluations of diaphragm function are easily acquired [9, 28]. Results from this study support the use of ultrasonography in diaphragm evaluation and integration in scanning protocols and implementation into daily practice may be relatively straight forward. Furthermore, evaluation of thickening fraction, which is highly relevant when weaning patients from mechanical ventilation, is not obtained by fluoroscopy only allowing evaluation of diaphragm excursion [5].

In conclusion, we found that the correlation between fluoroscopy and M-mode ultrasonography during an IC inspiration manoeuvre is acceptably high to suggest replacement of fluoroscopy by ultrasonography in evaluation of diaphragm motion.

### Supplementary Information

**Additional file 1: Figure S1.** Histogram of hemidiaphragm motion measured by fluoroscopy (boxes) and M-mode (droplines). **Table S1.** Mean hemidiaphragm motion measured by fluoroscopy in IC inspiratory capacity. **Table S2.** Contrast between repeated fluoroscopy measurements during inspiratory capacity. **Table S3.** Mean hemidiaphragm motion measured by fluoroscopy in sniff inspiration. **Table S4.** Contrast between repeated fluoroscopy measurement during sniff inspiration.

## Data Availability

All data are available for sharing upon contact with the corresponding author.
