# Supplementary material for: Replacement of fluoroscopy by ultrasonography in the evaluation of hemidiaphragm function, an exploratory prospective study
Source: Ultrasound J. 2024 Jan 8;16:1. doi: 10.1186/s13089-023-00355-0 (PMC10774234; doi:10.1186/s13089-023-00355-0)
Supplement: Supplementary file 1 — Additional file 1: Figure S1. Histogram of hemidiaphragm motion measured by fluoroscopy (boxes) and M-mode (droplines). Table S1. Mean hemidiaphragm motion measured by fluoroscopy in IC inspiratory capacity. Table S2. Contrast between repeated fluoroscopy measurements during inspiratory capacity. Table S3. Mean hemidiaphragm motion measured by fluoroscopy in sniff inspiration. Table S4. Contrast between repeated fluoroscopy measurement during sniff inspiration. [file 13089_2023_355_MOESM1_ESM.docx]

Additional file 1

Figure S1: Histogram of hemidiaphragm motion measured by fluoroscopy (boxes) and M-mode (droplines).

| Inspiratory Capacity inspiration | | | | | | |
| --- | --- | --- | --- | --- | --- | --- |
|  | Mean | S.E | Z | P | 95%CI | |
| 1 | 3.53 | 0.63 | 5.59 | 0.00 | 2.29 | 4.77 |
| 2 | 4.84 | 0.40 | 12.04 | 0.00 | 4.05 | 5.62 |
| 3 | 3.98 | 0.35 | 11.48 | 0.00 | 3.30 | 4.66 |
| 4 | 4.15 | 0.35 | 11.96 | 0.00 | 3.47 | 4.83 |
| 5 | 4.07 | 0.35 | 11.59 | 0.00 | 3.38 | 4.76 |

Table S1: Mean hemidiaphragm motion measured by fluoroscopy in IC inspiratory capacity.

| Inspiratory Capacity inspiration | | | | | | | |
| --- | --- | --- | --- | --- | --- | --- | --- |
| time | | Contrast | S.E | Z | P | 95%CI | |
| 2 | vs 1 | 1.31 | 0.60 | 2.20 | 0.03 | 0.14 | 2.48 |
| 3 | vs 1 | 0.45 | 0.56 | 0.80 | 0.42 | -0.65 | 1.54 |
| 4 | vs 1 | 0.63 | 0.56 | 1.12 | 0.26 | -0.47 | 1.72 |
| 5 | vs 1 | 0.54 | 0.56 | 0.96 | 0.34 | -0.56 | 1.64 |
| 3 | vs 2 | -0.86 | 0.27 | -3.13 | 0.00 | -1.40 | -0.32 |
| 4 | vs 2 | -0.68 | 0.28 | -2.48 | 0.01 | -1.22 | -0.14 |
| 5 | vs 2 | -0.77 | 0.28 | -2.75 | 0.01 | -1.32 | -0.22 |
| 4 | vs 3 | 0.18 | 0.19 | 0.95 | 0.34 | -0.19 | 0.54 |
| 5 | vs 3 | 0.09 | 0.19 | 0.47 | 0.64 | -0.29 | 0.47 |
| 5 | vs 4 | -0.09 | 0.19 | -0.45 | 0.66 | -0.46 | 0.29 |

Table S2: Contrast between repeated fluoroscopy measurements during inspiratory capacity.

| Sniff inspiration | | | | | | |
| --- | --- | --- | --- | --- | --- | --- |
|  | Mean | S.E | Z | P | 95%CI | |
| 1 | 2.02 | 0.19 | 10.69 | 0.00 | 1.65 | 2.40 |
| 2 | 2.12 | 0.21 | 10.21 | 0.00 | 1.72 | 2.53 |
| 3 | 1.57 | 0.19 | 8.26 | 0.00 | 1.20 | 1.95 |
| 4 | 1.77 | 0.20 | 8.99 | 0.00 | 1.39 | 2.16 |
| 5 | 1.75 | 0.19 | 9.02 | 0.00 | 1.37 | 2.13 |

Table S3: Mean hemidiaphragm motion measured by fluoroscopy in sniff inspiration.

| Sniff inspiration | | | | | | |
| --- | --- | --- | --- | --- | --- | --- |
| time | Contrast | S.E | Z | P | 95%CI |  |
| 2 vs 1 | 0.10 | 0.18 | 0.56 | 0.58 | -0.25 | 0.44 |
| 3 vs 1 | -0.45 | 0.15 | -2.92 | 0.00 | -0.75 | -0.15 |
| 4 vs 1 | -0.25 | 0.16 | -1.55 | 0.12 | -0.57 | 0.07 |
| 5 vs 1 | -0.27 | 0.16 | -1.73 | 0.08 | -0.58 | 0.04 |
| 3 vs 2 | -0.55 | 0.18 | -3.11 | 0.00 | -0.89 | -0.20 |
| 4 vs 2 | -0.35 | 0.18 | -1.90 | 0.06 | -0.71 | 0.01 |
| 5 vs 2 | -0.37 | 0.18 | -2.06 | 0.04 | -0.72 | -0.02 |
| 4 vs 3 | 0.20 | 0.16 | 1.22 | 0.22 | -0.12 | 0.52 |
| 5 vs 3 | 0.18 | 0.16 | 1.11 | 0.27 | -0.14 | 0.49 |
| 5 vs 4 | -0.02 | 0.17 | -0.13 | 0.90 | -0.35 | 0.30 |

Table S4: Contrast between repeated fluoroscopy measurement during sniff inspiration.
